# Analysis on vaccine hesitation and its associated factors among parents of preschool children in Songgang Street, Shenzhen

**DOI:** 10.1038/s41598-022-12530-9

**Published:** 2022-06-08

**Authors:** Xin Shen, Xia Wu, Zhenyu Deng, Xiang Liu, Yi Zhu, Yuchai Huang, Yuhua Deng, Qingfeng Tian, Yong Gan, Yanhong Gong, Zuxun Lu

**Affiliations:** 1 Department of Social Medicine and Health Management, School of Public Health, Tongji Medical College, Huazhong University of Science and Technology, No. 13 Hangkong Road, Wuhan, 430030 Hubei China; 2 Songgang People’s Hospital, Shenzhen, Guangdong China; 3 Department of Social Medicine and Health Management, College of Public Health, Zhengzhou University, Zhengzhou, Henan China

**Keywords:** Health care, Risk factors

## Abstract

China has the largest number of vaccinated population around the world. However, there has been few research on the prevalence and associated factors of vaccine hesitation among parents of preschool children. Therefore, we conducted this study to evaluate the status of vaccine hesitation and its associated factors among children’s parents. A cluster random sampling method was adopted to select six community health service centers in Shenzhen, and parents of preschool children who were immunized in the vaccination outpatient department of the selected community health centers were surveyed using a structured self-administered questionnaire. Vaccine hesitation was assessed by the Parent Attitudes about Childhood Vaccines (PACV) scale. A multiple linear regression analysis was used to assess the associated factors for vaccine hesitance among children's parents. A total of 1025 parents (response rate, 93.18%) filled out the questionnaires. The average score of vaccine hesitancy was 43.37 (SD = 10.34) points. 23.61% of parents wanted children to get all the recommended shots, 53.76% of them did not believe that many of the illnesses shots prevent were severe, and 75.41% of them could not guarantee the information they receive about shots. The results of multiple linear regression showed that the number of children in the family (*β* = −0.93, 95% CI: −1.31 to 0.54), health status of the child (*β* = 0.47, 95% CI: 0.07 to 0.87), education level of the parents (Father: *β* = −0.84, 95%CI: −1.37 to 0.31; Mother: = −1.59, 95%CI:−2.13 to −1.05), and annual family income (*β* = 1.64, 95%CI: 1.13–2.16) were significantly associated with vaccine hesitation. The average score of parents' vaccine hesitation in Shenzhen was 43.37. The results showed that the number of children in the family, health status of the children, education level of the parents and annual family income were important factors associated with the parents' vaccine hesitation.

## Introduction

Vaccine is one of the most effective measures to prevent infectious diseases, which saves more than three million deaths and prevents 750 thousand children from disability worldwide each year^1^. Since the invention of the first vaccine in the seventeenth century, more than twenty kinds of vaccines have been gradually applied to prevent diseases^2^.With the spread of COVID-19 around the world, great efforts were taken to develop vaccines against it, which also implied that vaccines play a pivotal role in the control of infectious diseases^3^.

In recent years, many researches have been carried out to improve vaccination rates worldwide. Vaccine hesitation, as an important aspect, received a lot of attention. Vaccine hesitancy is different from vaccination denial, which is somewhere in between total anti-vaccine and total pro-vaccine, and varies across time^4^. In 2012, vaccine hesitancy was defined by the World Health Organization (WHO) as a delay in acceptance or refusal of vaccination despite availability of vaccination services^5,6^.

According to the report of Centers for Disease Control of the United States, a quarter to a third of American children were affected by vaccine hesitancy^7^. A study conducted by Larson et al. reported that vaccine hesitation was particularly pronounced in Europe, where the rate of vaccine hesitation was 45.20% in France^8^. Jessica et al. ^9^ found that 43.00% of parents of children in Australia heavily concerned about vaccine efficacy, and approximately 25% of parents lacked confidence in vaccines. In addition, previous studies have shown that there were a few factors associated with vaccine hesitancy. A study by MacDonald et al.^4^ showed that age, sex, and place of residence were the influencing factors for vaccine hesitancy. Another study by Jacobson et al.^10^ showed that the number of children in one family and health status of children had a significant impact on parental vaccine hesitancy. This study by Xiao et al.^11^ showed that parents' education and income level were also potential influencing factors. Final, Williams^12^ found that the role of the primary caregiver of the children and time spent with children per day were possible associated with parents’ vaccine hesitancy.

China has the largest number of vaccination in the world each year^13^. According to the report by the Chinese Center for Disease Control, a total of 536 million vaccinations were administered in 2019^13,14^. The Chinese government has attached great importance to vaccination. Since 1978, it has been implemented the National Program for Immunization of Children, and has established a complete vaccine chain and a strict vaccine management system^15^. However, there has been few research on the prevalence and associated factors of vaccine hesitation among Chinese parents of children. Rend et al.^16^ and Wagner et al.^17^ investigated the vaccine hesitancy among parents of infants and children (age < 18 years) in Shanghai, respectively. However, Wang et al.^17^ conducted a study to examine childhood vaccination delay, explore the association between vaccination delay and parental vaccine hesitancy, and assess childhood vaccination delays during the coronavirus disease 2019 (COVID-19) pandemic in Wuxi, China. However, our focused on the prevalence and associated factors of parental vaccine hesitancy in Songgang Street, Shenzhen. This study aimed to investigate the current situation of vaccine hesitation among Shenzhen parents of preschool children based on a cross-sectional survey, and to explore its influencing factors. This study will fill in the shortage of data on vaccine hesitancy, and provide an important basis for the progress of vaccination planning.

## Results

### Characteristics of participants

The average age of the mothers and fathers were 29.60 (SD = 4.70) and 31.70 (SD = 5.00) years, respectively. About 30% of children’s mothers and fathers had primary school or below. Less than half (44.10%) of respondents s annual family income ranged from 100 to 200 thousand RMB. Among them, the majority (80.00%) lived in rural areas and approximately 70% migrated from other provinces. More than 75% children were cared by their mothers (76.30%). The basic information of children and their parents is shown in Table 1.

**Table 1:** Study characteristics of children and their parents.

Variables	N (%)
**Children**	
Age group, y	
< 1	321(31.30)
1 ~ 2	454 (44.30)
≧3	250 (24.40)
Sex	
Male	522 (50.90)
Female	503 (49.10)
Census registering	
Census register cities	141 (13.80)
Census register provinces and other cities	183 (17.90)
Census register other provinces	701 (68.40)
Place of residence	
Urban	205 (20.00)
Rural	820 (80.00)
Number of children	
One child	464 (45.30)
More than one child	561 (54.70)
Health status	
Good	530 (51.70)
Fair	145 (14.10)
Poor	350 (34.10)
**Parents**	
Age group of mothers, y	
< 28	355 (34.60)
28 ~ 32	412 (40.20)
> 32	258 (25.20)
Mothers’ education level	
Primary school or below	361 (35.20)
Middle school	418 (40.80)
College degree or above	246 (24.00)
Age group of fathers, y	
< 30	353 (34.40)
30 ~ 33	346 (33.80)
> 33	326 (31.80)
Fathers’ education level	
Primary school or below	317 (30.90)
middle school	418 (40.80)
College degree or above	290 (28.30)
The primary caregiver of children	
Father	30 (2.90)
Mother	782 (76.30)
Maternal/Grandparent	183 (17.90)
Other	30 (2.9)
Time spent with children per day (hours)	
< 4	191 (18.60)
4 ~ 6	253 (24.70)
> 6	581 (56.70)
Annual family income (Ten thousand RMB)	
< 10	334 (32.60)
10 ~ 20	452 (44.10)
20	239 (23.30)

### Survey findings

The average score of vaccine hesitation was 43.37 (standard deviation, SD = 10.34). Responses to the individual items that make up the PACV scale are shown in Table 2.

**Table 2:** Current situation of vaccine hesitation among parents of preschool children.

	Items	Agree (%)	Uncertain (%)	Disagree (%)
Behaviors	Q1: Have you ever delayed having your child get a shot for reasons other than illness or allergy?	71.22	17.95	10.83
	Q2: Have you ever decided not to have your child get a shot for reasons other than illness or allergy?	74.73	15.32	9.95
General Attitudes	Q3: How concerned are you that a shot might not prevent the disease?	25.56	40.68	33.76
	Q4: If you had another infant today, would you want him/her to get all the recommended shots?	23.61	38.83	37.56
	Q5: How sure are you that following the recommended shot schedule is a good idea for your child?	0.20	42.63	57.17
	Q6: Children get more shots than are good for them	1.37	14.83	83.80
	Q7: I believe that many of the illnesses shots prevent are severe	0.68	45.56	53.76
	Q8: Overall, how hesitant about childhood shots would you consider yourself to be?	14.24	53.37	32.39
	Q9: I trust the information I receive about shots	4.88	19.71	75.41
	Q10: I am able to openly discuss my concerns about shots with my child's doctor	43.02	42.15	14.83
	Q11: All things considered, how much do you trust your child's doctor?	22.63	35.41	41.95
Safety and Efficacy	Q12: It is better for my child to develop immunity by getting sick than to get a shot	7.41	48.88	43.71
	Q13: It is better for children to get fewer vaccines at the same time	85.17	3.71	11.12
	Q14: How concerned are you that your child might have a serious side effect from a shot?	1.46	19.51	79.02
	Q15: How concerned are you that anyone of the childhood shots might not be safe?	58.05	4.68	37.27

With regard to vaccination behaviors, 71.22% of parents have delayed to have their child get a shot for reasons other than illness or allergy, and 74.73% of parents decided not to have their child get a shot for reasons other than illness or allergy.

In the aspect of general attitudes with vaccine, 25.56% of parents reported their concern that a shot might not prevent the disease. 23.61% of parents wanted children to get all the recommended shots, 53.76% of parents hesitated to believe that many of the illnesses shots prevent are severe, 75.41% of parents could not guarantee the information they receive about shots.

For vaccine safety and efficacy, 79.02% of parents concerned that their child might have a serious side effect from a shot in the item 14. 37.27% of parents worried that anyone of the childhood shots might not be safe. 85.17% of parents believed it is better for children to get fewer vaccines at the same time.

### Influencing factors of parents' vaccine hesitation

Table 3 presents the results of the multiple linear regression model analysis. The fitted model was statistically significant (*P* < 0.001). The results showed that parents' vaccine hesitation was associated with five factors including the number of children in the family, the health status of the child, the education level of the parents (both father and mother), and the annual family income.

**Table 3:** Multiple linear regression analysis of influencing factors of parents’ vaccine hesitation.

Variables	Unstandardized Coefficients		Standardized Coefficients	*t*	*p*	95%CI
	*β*	*SE*	*β*			
Constant	**13.82**	**0.71**	**NA**	**19.58**	**< 0.001**	**12.44–15.20**
**Children**						
Age group, y (Ref: < 1)						
1 ~ 2	0.37	0.21	0.06	1.72	0.09	−0.05 to 0.78
≥ 3	0.45	0.25	0.06	1.81	0.07	−0.04 to 0.94
Gender (Ref: Male)						
Female	−0.01	0.18	0.01	−0.01	0.99	−0.36 to 0.36
Census register (Ref: Census register city)						
Census register province and other cities	0.04	0.42	0.01	0.09	0.93	−0.78 to 0.86
Census register other provinces	−0.14	0.40	−0.02	−0.36	0.72	−0.92 to 0.64
Place of residence (Ref: Urban)						
Rural	0.25	0.34	0.03	0.74	0.46	−0.41 to 0.91
Number of children (Ref: One child)						
More than one child	**−0.93**	**0.20**	**−0.15**	**−4.68**	** < 0.001**	**−1.31 to −0.54**
Self-reported health status (Ref: Good)						
Fair	−0.05	0.28	−0.01	−0.16	0.87	−0.58 to 0.49
Poor	**0.47**	**0.20**	**0.07**	**2.31**	**0.02**	**0.07 to 0.87**
**Parents**						
Age group of mothers, y (Ref: < 28)						
28 ~ 32	−0.23	0.26	−0.04	−0.87	0.39	−0.73 to 0.28
> 32	0.46	0.37	0.07	1.25	0.21	−0.26 to 1.19
Mothers’ education level (Ref: Primary school or below)						
Middle school	−0.12	0.23	−0.02	−0.51	0.61	−0.56 to 0.33
College degree or above	**−1.59**	**0.28**	**−0.22**	**−5.74**	** < 0.001**	**−2.13 to −1.05**
Age group of fathers, y (Ref: < 30)						
30 ~ 33	0.25	0.26	0.04	0.97	0.33	−0.26 to 0.76
> 33	0.18	0.34	0.03	0.52	0.60	−0.49 to 0.84
Fathers’ education level (Ref: Primary school or below)						
Middle school	**−0.54**	**0.23**	**−0.09**	**−2.29**	**0.02**	**−0.10 to −0.08**
College degree or above	**−0.84**	**0.27**	**−0.12**	**−3.09**	**0.01**	**−1.37 to −0.31**
The primary caregiver of children (Ref: Father)						
Mother	−0.56	0.54	−0.08	−1.02	0.31	−1.62 to 0.51
Maternal/Grandparent	0.04	0.58	0.01	0.06	0.95	−1.10 to 1.17
Other	1.25	0.75	0.07	1.66	0.10	−0.22 to 2.72
Time spent with children per day (hours) (Ref: < 4)						
4 ~ 6	0.04	0.29	0.01	0.12	0.90	−0.53 to 0.60
> 6	−0.18	0.27	−0.03	−0.69	0.50	−0.70 to 0.34
Annual family income (Ten thousand RMB) (Ref: < 10)						
10–20	0.18	0.21	0.03	0.84	0.40	−0.24 to 0.60
20	**1.64**	**0.26**	**0.23**	**6.25**	** < 0.001**	**1.13 to 2.16**

Significance values are in bold.

Vaccine hesitation among parents of child who was not the first child in the family was lower than that of those who had only one child (*β* = −0.93, 95%CI: −1.31 to −0.54). Parents of child who have poor health status had higher vaccine hesitation score than that of those whose child had good health status (*β* = 0.47, 95%CI: 0.07–0.87). The vaccine hesitation scores of parents with high financial status were higher compared with those with poor financial status (*β* = 1.64, 95%CI: 1.13–2.16). Compared with parents who had primary school of below, parents who had achieved a bachelor degree or above had lower vaccine hesitation scores (Father: *β* = −0.84, 95%CI: −1.37 to −0.31; Mother: *β* = −1.59, 95%CI: −2.13 to −1.05).

## Discussion

As the decision makers of childhood immunization, parents' cognition and attitude about childhood vaccines have significant influence on their vaccine hesitation. Given the serious consequences of vaccine hesitation, it has attracted global concern in recent years. In order to solve the problem of vaccine hesitation, it is necessary to understand the current situation of vaccine hesitation among parents of child and clarify its influencing factors. This study showed that parents had a high level of vaccine hesitation in Shenzhen, China, and parents’ vaccine hesitation was significant associated with the number of children in the family, health status of children, education level of the parents, and annual family income.

The WHO Strategic Advisory Group of Experts (SAGE) Report from the Working Group on vaccine hesitancy showed that 93% of WHO member states reported vaccine hesitation, which indicated that vaccine hesitation was a globally social problem^18^. The results of this study showed that most Chinese parents had vaccine hesitation, although there was a difference in response. Vaccine hesitancy is different from vaccination denial, which is somewhere in between total anti-vaccine and total pro-vaccine, and varies across timeFor example, Qing He et al.^19^ found that 33.04% of parents were hesitant about vaccines one week after the vaccine safety accident occurred, but the percentage decreased six months later. In addition, vaccine hesitation was influenced by policy, culture, and religion, as well as measurement criteria of vaccine hesitant. It was certain that vaccine hesitant was common around the world^18^. For example, Alexander et al. examined global trendy of vaccine confidence using data from 290 surveys across 149 countries in 2015–2019^20^ and found that confidence in the importance, safety, and effectiveness of vaccine decreased in Afghanistan, Indonesia, Pakistan, Philippines, and South Korean.

For influencing factors of vaccine hesitation, study of Metin et al.^21^ and Jessica et al.^22^ shown that parents' education level had an important impact on their vaccine hesitation. Previous studies conducted in Nigeria, India and Kyrgyzstan indicated that parents' lower education level was an obstacle to their children's vaccination^23,24^. Similarly, previous studies from the United States, Greece and the Netherlands had found that parents’ higher education was a promoter of vaccination^15,21,25–27^. This study also found the significant association between parents' educational background and their vaccine hesitation. To be specific, Chinese parents' vaccine hesitation decreased with the improvement of their educational background. A prior qualitative study showed that highly educated parents believed in their ability to make correct vaccination decisions based on their higher education level and self-sourced vaccination information^28^. Family income has also been identified in previous studies as an important factor affecting vaccine acceptance. Studies from the United States and Nigeria have shown that low family income is a barrier to vaccination services^29,30^. However, this study showed that parents with higher income levels scored higher on vaccine hesitation, which was inconsistent with the results from the United States and Nigeria. This could be due to that richer families are more likely to downplay the ill effects of illness.

In addition to parental factors, this study also found that factors such as the number of children and the health status of children were also significant associated with parental vaccine hesitation. To be specific, if the child was the first child in the family, parents’ hesitation degree would increase; by contrast, if the child was not the first child, parents has a stronger willingness to vaccinate their children, which may be associated with their vaccination experience.

Studies found that parents realized their responsibility in their children's health, but the responsibility was associated with anxiety and fear; thus, they were very cautious in vaccination for their children. One possible explanation was that parents worried that vaccination may hurt their children^31,32^. The results of this study confirmed this view, that is to say, the worse the health status of the vaccinated children, the higher the level of their parents' vaccine hesitation. Parental behaviors of vaccine rejection or vaccine hesitation was complex and multifaceted. It should be noted that concern about the efficacy and safety of vaccination among parents of preschool children should be considered in policy-making.

### Strength and limitations

In 2020, more than 536 million vaccines were administered in China. As a country with a maximum of vaccination for children, China had few researches on preschool children parents' vaccine hesitation. This is the first study to be carried out in Shenzhen. Although this study was a cross-sectional study in cities, it recruited the largest number of participants and described parents’ vaccine hesitation comprehensively. This study can precisely reflect the current statuses of vaccine hesitant among parents of preschool children, which will be helpful to improve vaccination program, and provide valuable results regarding vaccine hesitant internationally.

The limitation of this study was that the potential influencing factors for vaccine hesitancy among parents could be more than those included in the questionnaire. The respondents we included in the study were all parents of preschool children, but may not be representative of all parents of preschool children, because some parents may choose hospitals or other medical institutions to vaccinate their children. Secondly, this was a cross-sectional study design, which precluded evaluation of temporality and causality of the observed relationships. Additional studies could consider conducting a community-based survey to evaluate the situation of parents' vaccine hesitation more comprehensively in China.

## Conclusions

The average score of parents' vaccine hesitation in Shenzhen was 43.37. The number of children in the family, health status of the children, education level of the mother/father, and annual family income were significant associated with parents' vaccine hesitation. Appropriate interventions are recommended to alter parents' hesitation about vaccines and ultimately ensure the sustainable development of immunization programme.

## Methods

### Ethics statement

This study was approved by the Research Ethics Committee of Tongji Medical College, Huazhong University of Science and Technology, Wuhan, China and performed in accordance with the principles of the Declaration of Helsinki. Respondents were informed that their participation was voluntary, and consent was implied on the completion of the questionnaire.

### Survey instrument

In the present study, vaccine hesitation was measured using the Parent Attitudes about Childhood Vaccines (PACV) scale, which was developed by Opel et al. PACV was a 15-item scale that assessed the vaccine hesitation in three dimensions, including the vaccination behaviors, immunization safety and beliefs on vaccine safety and efficacy, and the attitudes on vaccine mandates. Each item has three answers including "agree", "uncertain" and "disagree", coded with value of "2", "1" and "0", respectively. The total score was computed as the sum of all items, ranging from 0 to 30, with higher scores indicating higher levels of vaccine hesitation. We converted this raw score to a 0–100 scale using simple linear transformation^33^. In previous studies, this scale has been applied to the Chinese population, and its reliability in the Chinese population has been verified^34^. The scale has shown good internal consistency (Cronbach’s α for the 3 sub-scale were 0.72, 0.83, and 0.77, respectively^35^.

In addition, information about participants' sociodemographic characteristics including age, sex, education, monthly family income, number of children, and age, sex, and health status of the youngest children were also collected. These factors mainly come from the previous literature^4,9–11^ and consultation with experts in related fields.

### Study population

This was a cross-sectional study and data were collected from October to December 2019 in the city of Shenzhen, Guangdong Province (Southern China). A cluster random sampling method was adopted in the present study. First, six community health service centers were randomly selected from the twelve community health service centers affiliated to the Song-gang People's Hospital in Shenzhen, which covered more than 600 thousand population in Song-gang Street. In our study, a 95% confidence level and ± 5% precision are assumed for the Equation.

$${\text{n = }}\frac{N}{{1 + N\mathop {(e)}\nolimits^{2} }}$$

where n is the sample size, N is the population size, and e was the level of precision. Thus, the conservative total sample size for this questionnaire was 400.

Second, all parents of preschool children who were immunized in the vaccination outpatient department at the study sites during the study period were recruited. A total of 1100 parents of children were enrolled and filled out a structured self-administered questionnaire, and 1025 valid questionnaires were collected with effective response rate of 93.18%. We noted in the questionnaire: Good health status meant that children adopt to various changes in the environment, had no general diseases, and had sufficient energy. Moderate health status meant that children were in an intermediate state between health and disease, and they experienced fatigue, weight loss, weakness and indigestion. Poor health was when a child was in a serious health-threatening condition such as infection, illness or malnutrition.

### Statistical analysis

*SPSS 25.0* was applied to analyze the data. Descriptive analyses included means for continuous variables and percentages for categorical data. A multiple linear regression model was used to examine the associations of independent variables with parents’ vaccine hesitation scores. All comparisons were two-tailed and a *P* value of < 0.05 was defined as statistically significant.

### Ethics approval and consent to participate

This study protocol was approved by the institutional review board of Tongji Medical College of Huazhong University of Science and Technology, Wuhan, China. Respondents were informed that their participation was voluntary, and informed consent was implied on the completion of the questionnaire.

## Data Availability

Data may be made available by contacting the corresponding author.
